# Supplementary material for: PPARα, δ and FOXO1 Gene Silencing Overturns Palmitate-Induced Inhibition of Pyruvate Oxidation Differentially in C2C12 Myotubes
Source: Biology (Basel). 2021 Oct 25;10(11):1098. doi: 10.3390/biology10111098 (PMC8614693; doi:10.3390/biology10111098)

Figure 1

PPARα

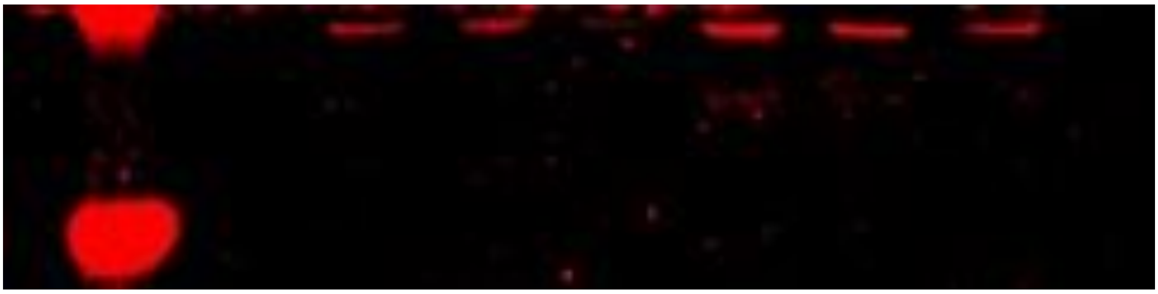

α-actin

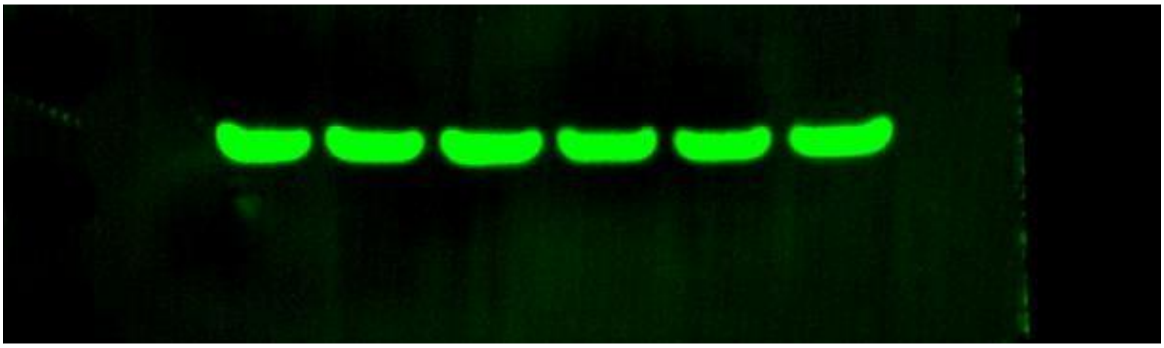

PPARδ

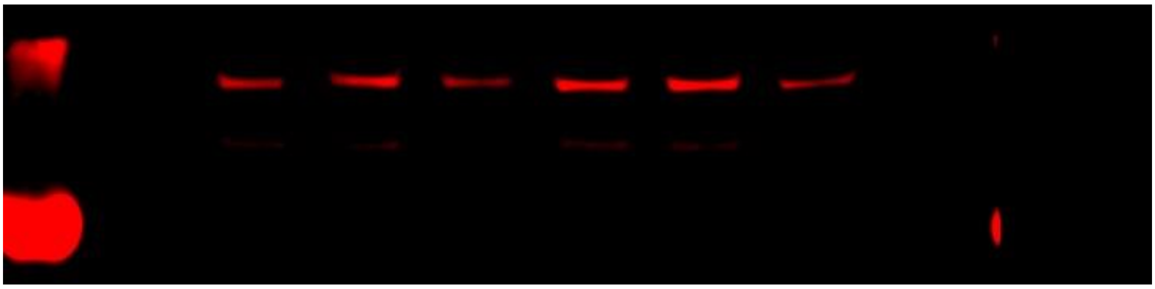

α-actin

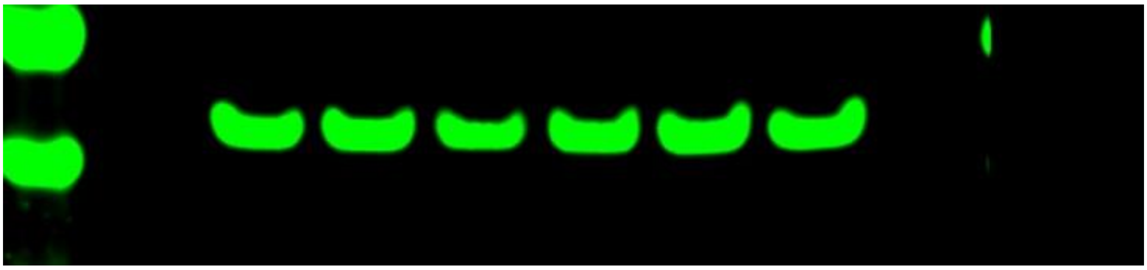

FOXO1

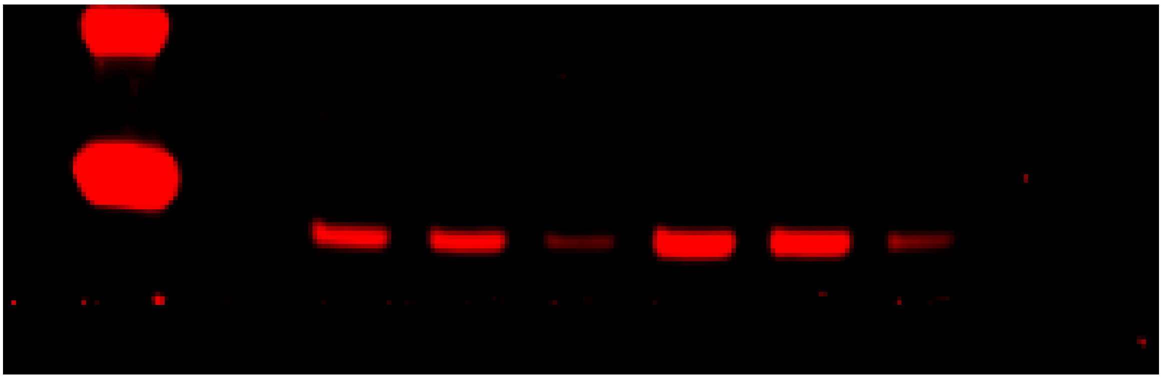

α-actin

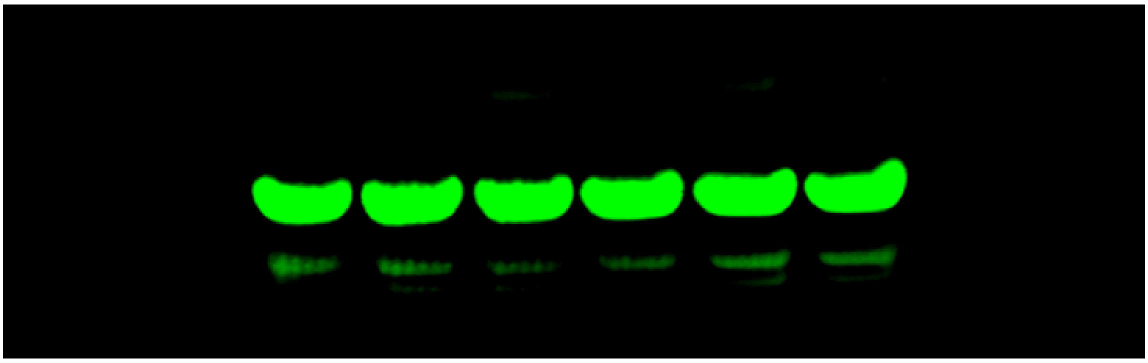

Figure 5

PDK4 (siPPARα)

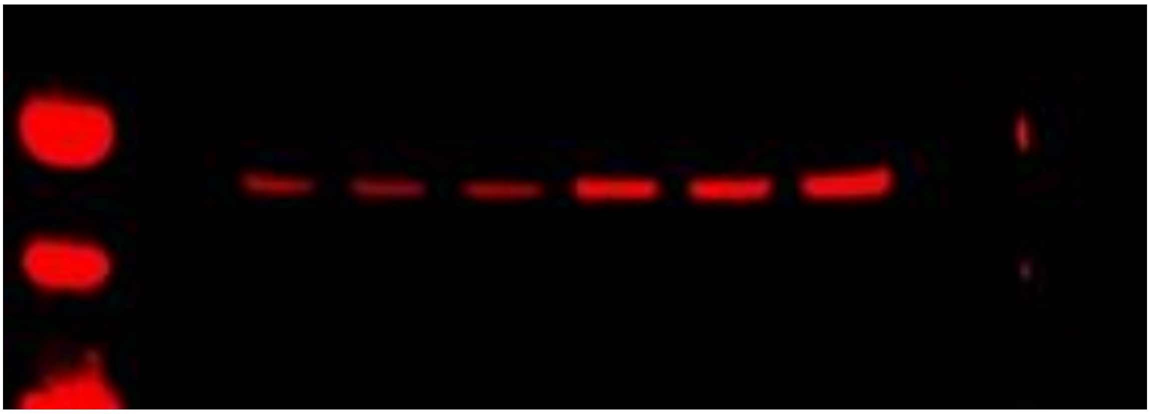

α-actin **(siPPARα)**

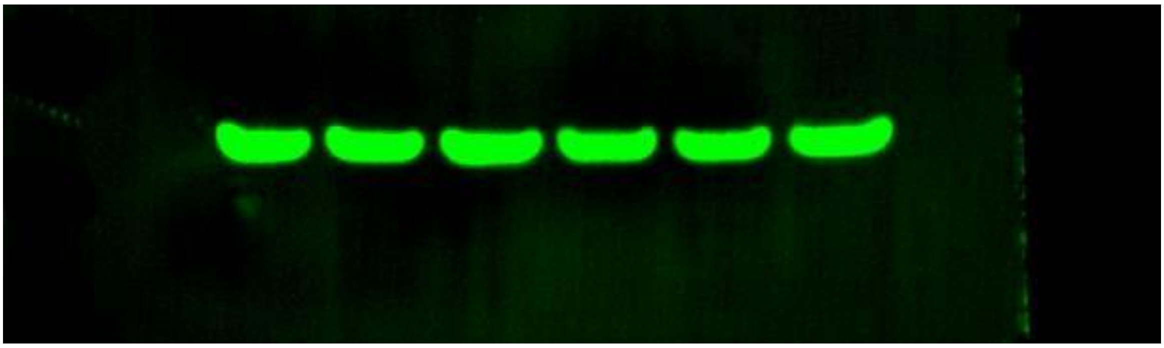

PDK4 (siPPARδ)

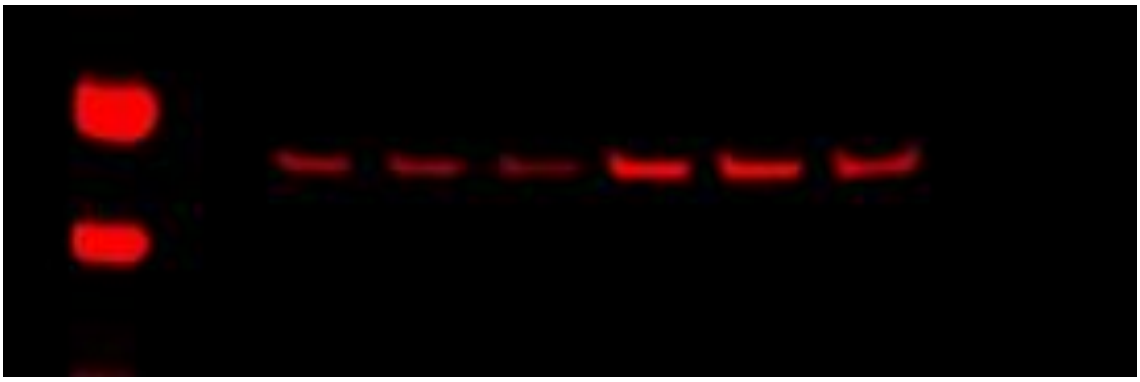

α-actin **(siPPARδ)**

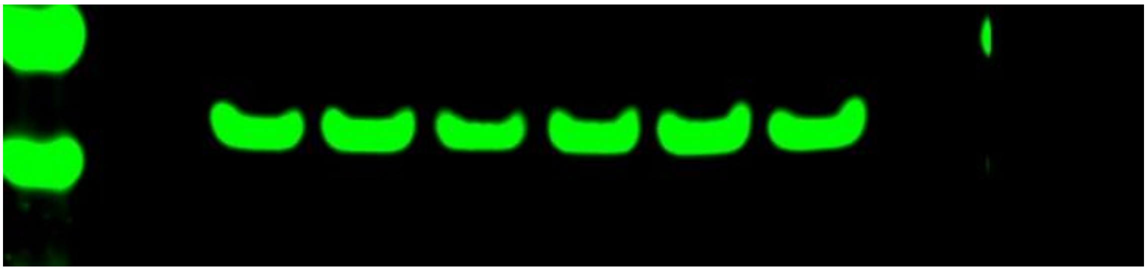

PDK4 (siFOXO1)

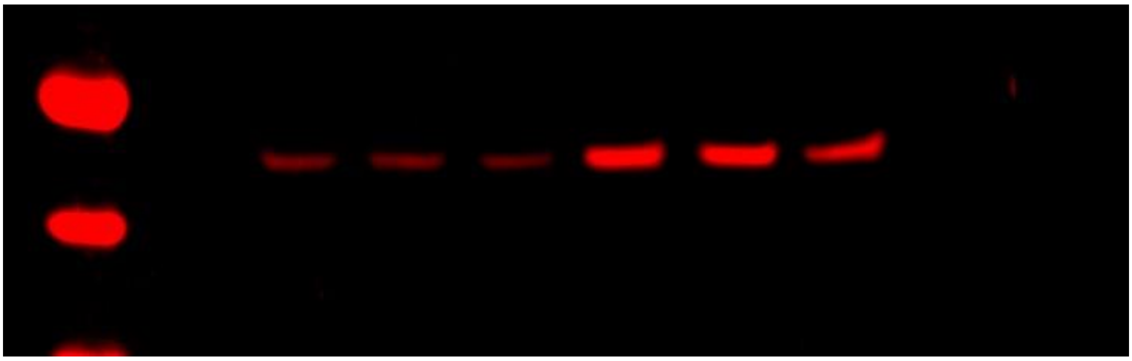

α-actin **(siFOXO1)**

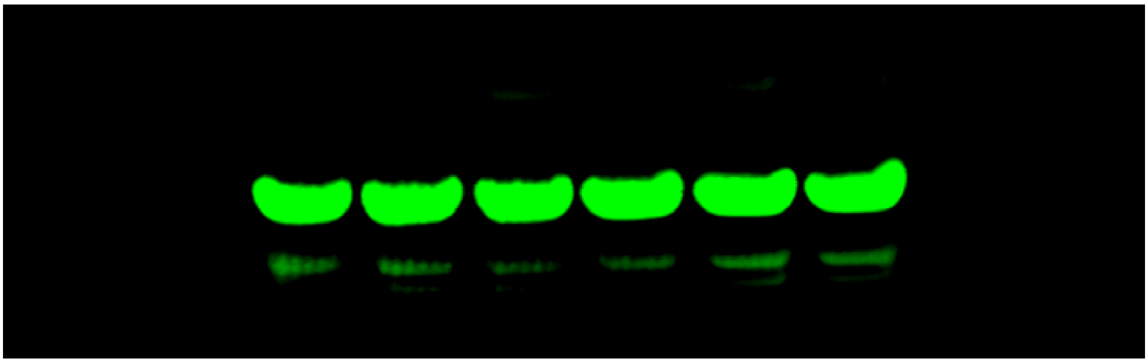

Figure 6

PPARδ

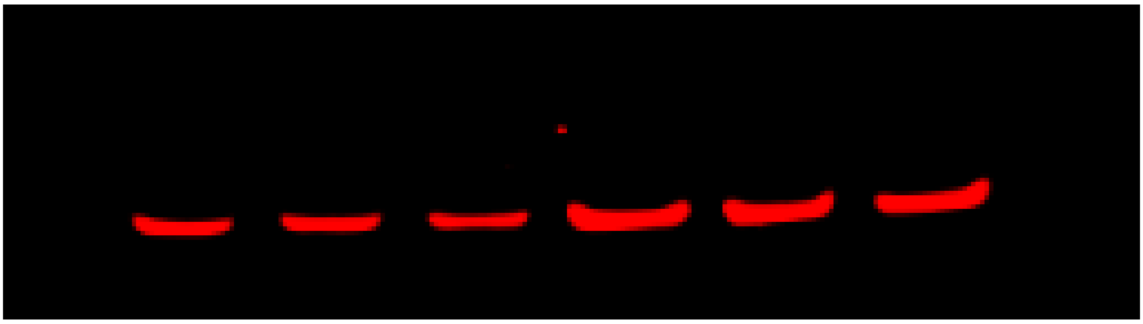

p-FOXO1

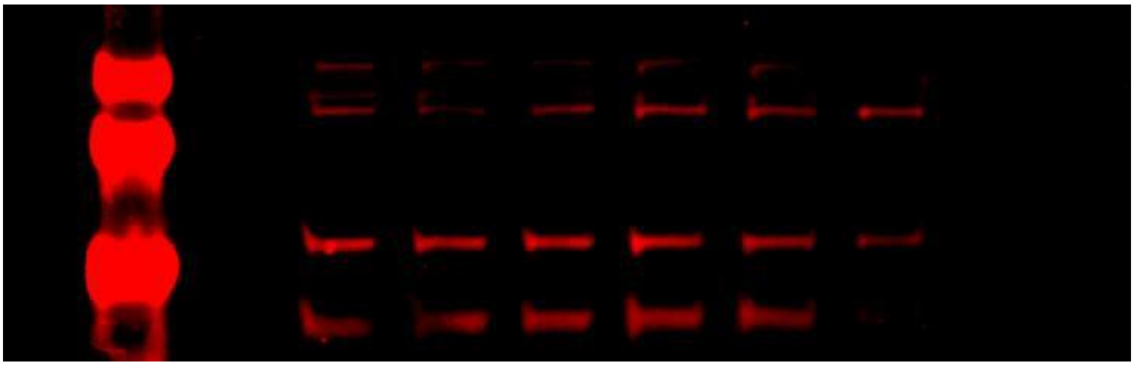

t-FOXO1

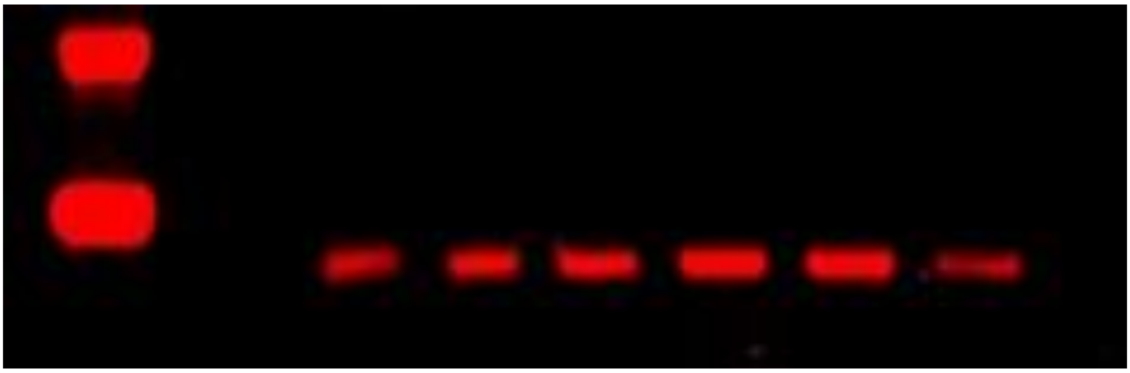

α-actin

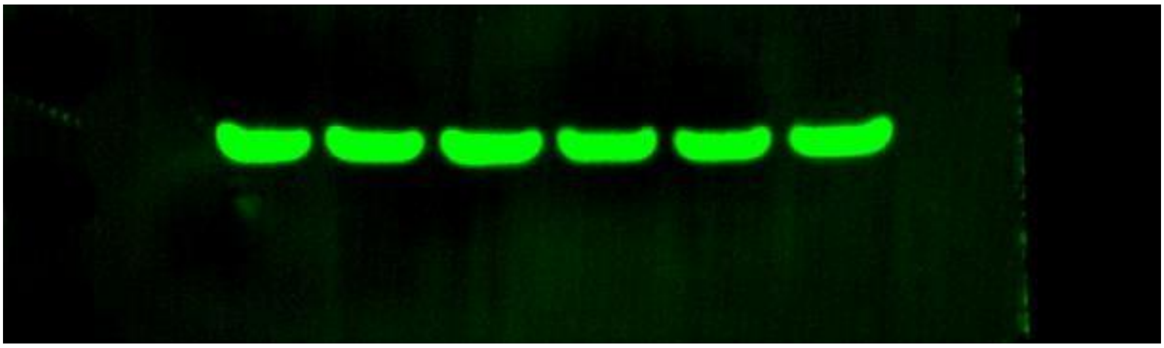

Figure 7

PPARα

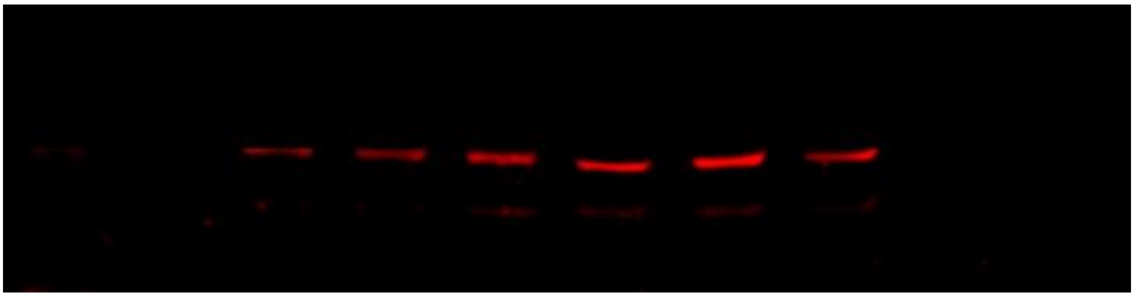

p-FOXO1

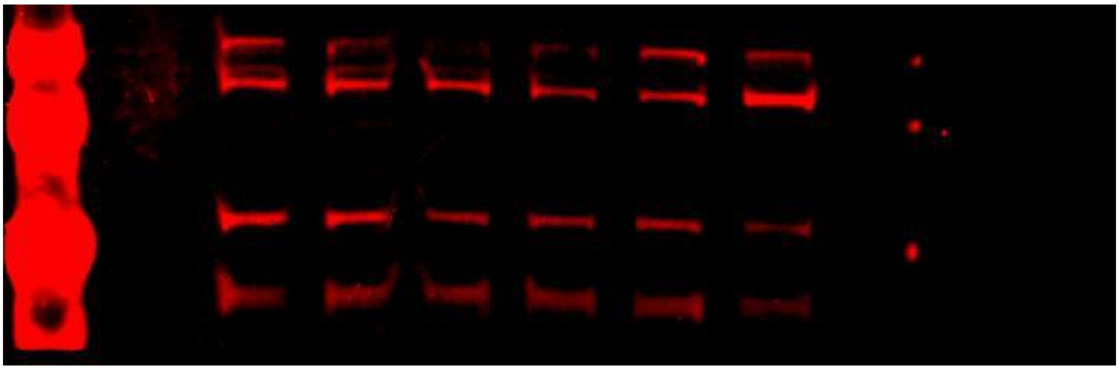

t-FOXO1

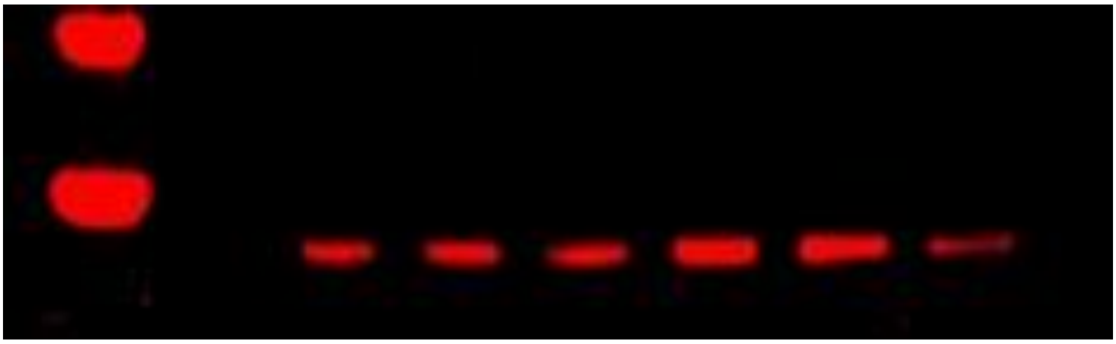

α-actin

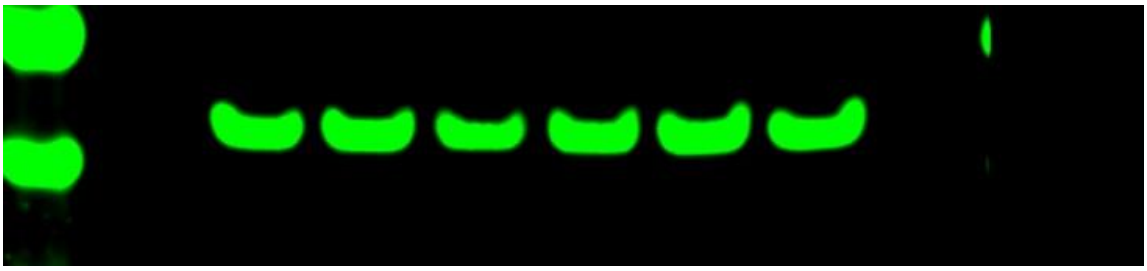

Figure 8

PPARα

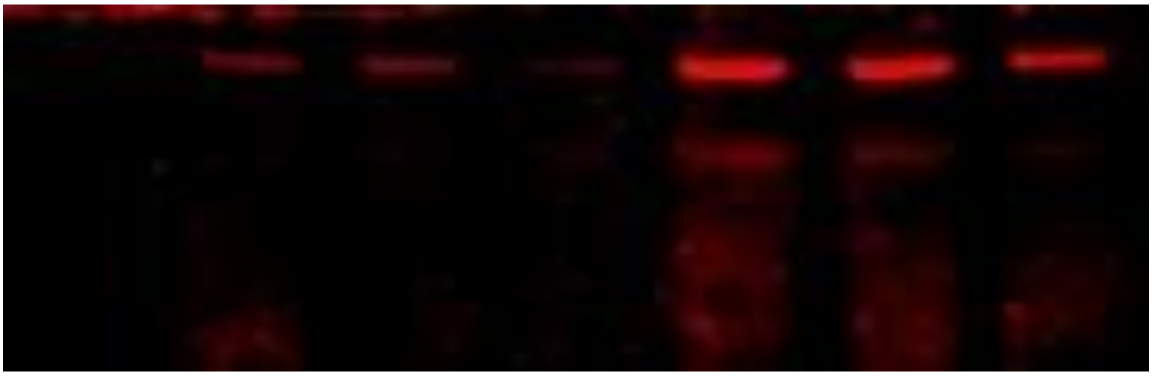

PPARδ

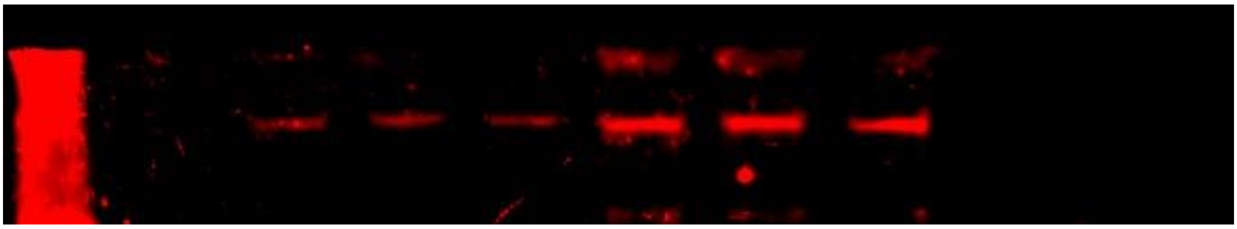

α-actin

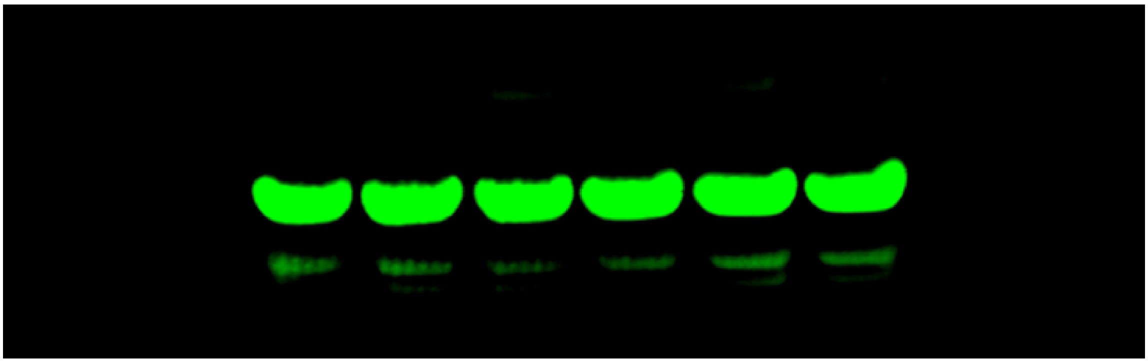

Supplement: Supplementary file 1 [file biology-10-01098-s001.zip › biology-1385626-original images-renumbered.pdf]
